# Associations between Physical Status and Training Load in Women Soccer Players

**DOI:** 10.3390/ijerph181910015

**Published:** 2021-09-23

**Authors:** Lillian Gonçalves, Filipe Manuel Clemente, Joel Ignacio Barrera, Hugo Sarmento, Gibson Moreira Praça, André Gustavo Pereira de Andrade, António José Figueiredo, Rui Silva, Ana Filipa Silva, José María Cancela Carral

**Affiliations:** 1 Faculty of Educational Sciences and Sports Sciences, University of Vigo, 36005 Pontevedra, Spain; chemacc@uvigo.es; 2 Escola Superior Desporto e Lazer, Instituto Politécnico de Viana do Castelo, Rua Escola Industrial e Comercial de Nun’Álvares, 4900-347 Viana do Castelo, Portugal; rui.s@ipvc.pt (R.S.); anafilsilva@gmail.com (A.F.S.); 3 Instituto de Telecomunicações, Delegação da Covilhã, 1049-001 Lisboa, Portugal; 4 Faculty of Sport Sciences and Physical Education, University of Coimbra, 3000-248 Coimbra, Portugal; jibarrera@outlook.es (J.I.B.); hg.sarmento@gmail.com (H.S.); afigueiredo@fcdef.uc.pt (A.J.F.); 5 Research Unit for Sport and Physical Activity (CIDAF), 3000-248 Coimbra, Portugal; 6 Sports Department, Universidade Federal de Minas Gerais, Belo Horizonte 31270-901, Brazil; gibson_moreira@yahoo.com.br (G.M.P.); andreguto@yahoo.com.br (A.G.P.d.A.); 7 The Research Centre in Sports Sciences, Health Sciences and Human Development (CIDESD), 5001-801 Vila Real, Portugal

**Keywords:** football, athletic performance, training load, sports training, physical fitness

## Abstract

This study aimed to analyze the variations of fitness status, as well as test the relationships between accumulated training load and fitness changes in women soccer players. This study followed an observational analytic cohort design. Observations were conducted over 23 consecutive weeks (from the preseason to the midseason). Twenty-two women soccer players from the same first Portuguese league team (22.7 ± 5.21 years old) took part in the study. The fitness assessment included anthropometry, hip adductor and abductor strength, vertical jump, change of direction, linear speed, repeated sprint ability, and the Yo-Yo intermittent recovery test. The training load was monitored daily using session rating of perceived exertion (s-RPE). A one-way repeated ANOVA revealed no significant differences for any of the variables analyzed across the three moments of fitness assessments (*p* > 0.05). The *t*-test also revealed no differences in the training load across the moments of the season (*t* = 1.216; *p* = 0.235). No significant correlations were found between fitness levels and accumulated training load (range: *r* = 0.023 to −0.447; *p* > 0.05). This study revealed no differences in the fitness status during the analyzed season, and the fitness status had no significant relationship with accumulated training load.

## 1. Introduction

Soccer is a high-intensity intermittent sport that recruits different energetic systems based on the intermittence of the match [1,2]. Among other factors, soccer performance requires technical skills, tactical awareness, and physical fitness [1,3]. In women’s soccer, players may cover 9–12 km in total in a single match, with 1.5–2.5 km covered during high-intensity runs [4,5,6]. Moreover, throughout a women’s soccer match, the average heart rate can reach up to 167 beats per minute (bpm), and the maximum heart rate (HRmax) can reach up to 193 bpm [7]. Therefore, to be successful, women soccer players should possess well-developed aerobic and anaerobic capacities, as well as good neuromuscular properties [2].

Well-developed physical fitness can help ensure overall success to the same extent as other important factors such as technical and tactical skills [1,8]. Accordingly, seeking an improvement in fitness status, it is necessary to understand the status of players, thus making the assessment a determinant factor for individualization of the training and controlling the development of the players [1,9]. Regarding the control of evolution, it is also expectable that some fitness variations may occur across a season, specifically considering the three main periods of training and competition: (i) preseason, (ii) early-season, and (iii) end-season [1,10,11].

For example, body fat is usually lower after the preseason training period than at the start of the preseason [10,12]. Additionally, significant changes occur in the biomarkers of physiological stress [1,13]. Considering the physical fitness of female soccer players, it was found that countermovement jump scores seem to improve during the season [1]. Furthermore, the linear speed at 15 m improves during the preseason before stabilizing until the end of the season, whereas the linear speed at 25 m starts to decrease at the end of the season [11]. Naturally, considering seasonal variations, most of the fitness changes occur during the preseason because the training sessions during this phase are focused on establishing the players’ fitness [3,14,15]. In contrast, during the season, more focus is placed on tactical and technical skills [16], with some efforts to stabilize players’ fitness.

Even though no perfectly related variations were observed across the season, physical/physiological adaptations could be related to the training load and stimuli imposed on the players [17]. Therefore, a dose–response relationship is expected to arise between the training load and changes in fitness that may occur in soccer players [18]. However, such a relationship can vary on the basis of the training load measures and fitness parameters used; moreover, the relationship might not be as obvious or straightforward as expected [18,19]. As an example, in a study conducted on professional soccer players, relationships were found between accumulated perceived exertion and the speed achieved in the 30–15 Intermittent Fitness Test by professional players [20]. However, in another study (also on soccer players), such a relationship was not meaningful [21].

As mentioned above, the magnitude of the relationship between load and adaptations can vary as a function of the measures used. In the case of training load monitoring, one of the most commonly used measures is the rating of perceived exertion (RPE) [22,23]. This measure has been confirmed as valid and reliable, based on different scales (e.g., CR-10, CR-100), to estimate the intensity of a training session. According to the score provided by the player, RPE can be used to calculate the session RPE (s-RPE), which is the multiplication of the RPE score by the duration of the session (in minutes) [23,24]. Since this measure (s-RPE) has been highly correlated with internal load markers (e.g., heart rate measures) and external load markers (e.g., total distance, player load) [25,26], it seems to be a good measure to test relationships with fitness adaptations across the season.

Adaptions in soccer players take time and are influenced by multiple factors, such as age, gender, training history, psychological factors, and the duration, intensity, and frequency of training [18,27]. Therefore, it is difficult to understand which factors promote changes in players in women’s soccer. In the particular case of women’s soccer, dose–response relationships have not been explored’ for that reason, there is a need to test whether such a relationship exists.

Testing the possibility of relationships between accumulated training load and the changes in fitness status would help to identify whether training load is a determinant of these changes or if there are other factors that coaches should be aware of. For that reason, the aims of this study were to analyze variations in the fitness status of women soccer players over time (repeated measures) and test the relationships between accumulated training load and fitness variations.

## 2. Materials and Methods

### 2.1. Experimental Approach

**Figure 1:**
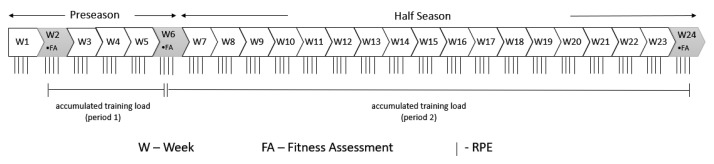
Timeline of the study.

This study followed an observational analytic cohort design. Observations were made across 23 consecutive weeks (from the preseason to midseason). Fitness assessments of the players were performed three times: (i) at the beginning of the preseason, (ii) at the end of the preseason, and (iii) during the middle of the season. Internal loads were collected daily in all training sessions between August and January (Figure 1).

### 2.2. Participants

The cohort included 22 female soccer players (age: 22.7 ± 5.21 years; height: 162 ± 6.84 cm; weight: 57.6 ± 4.9 kg) competing in the first Portuguese League. The team had four weekly training sessions and one official match per week. The eligibility criteria for being considered in the analysis were as follows: (i) participation in at least 85% of the training sessions during the study, (ii) participants were present in all three assessments, (iii) absence of injuries or illness in the last four consecutive weeks, and (iv) players had at least 2 years of experience. Three players were excluded because they did not participate in all physical assessments. Before the assessments, all players were informed about the study procedures and signed an informed consent. The study was approved by the local university and followed the ethical standards of the Declaration of Helsinki for the study of humans.

### 2.3. Fitness Assessment

Fitness assessments were conducted between August and January. All tests were performed during the same day of the week, following the same order, and at the same time of the day (7:30 p.m.) to limit data bias. During the three periods of assessments, all tests were distributed across three sessions, interspersed by 24 h of recovery. We acknowledge the fact that a testing battery can be carried out in a single day [28]. However, it can ideally be distributed over 2–3 days [29]. Regardless of the days, it is important that the sequence is designed with the aim of ensuring the most adequate conditions of absence of fatigue in tests with a greater need for neuromuscular recruitment, leaving the tests with greater metabolic stress to the end [29]. Bioenergetic and neuromuscular considerations resulted in the applied test sequencing in the present study. Regarding the warm-up protocol, it was out of the scope of the authors to intervene as it was always the team staff (physical trainer) conducting the warm-ups. The warm-ups consisted of low and self-paced running, followed by calisthenic exercises in which players performed two sets of 10 repetitions of walking lunges, single-leg deadlifts, and fontal and lateral high knee movements. These warm-ups were based on proposed strategies, highlighting the post-activation potentiation (PAP) exercises, as previously recommended and used [30,31].

The first assessments comprised anthropometry and hip adductor and abductor strength tests. The second assessments comprised lower-body power, change-of-direction (COD), and linear speed tests. The third assessments comprised repeated sprint ability (RSA) and Yo-Yo intermittent recovery (YYIR) tests. All indoor tests were performed in a room with a stable temperature of 23 °C and relative humidity of 55%. All field tests were conducted on a synthetic turf with a mean temperature of 19.5 ± 3.4 °C and a relative humidity of 63% ± 4%.

A measuring tape (SECA 206, Hamburg, Germany) and a digital scale (SECA 874, Hamburg, Germany) were used to measure the participants’ height and body weight, measured to the nearest 0.1 kg. During both assessments, all participants were in a vertical position and had no shoes and unnecessary accessories. To measure hip strength, the squeeze test was conducted using a dynamometer (Smart Groin Trainer, Neuro excellence, Portugal), as in a previously recommended protocol [32]. For lower-body power performance, the squat jump (SJ) and countermovement jump (CMJ) with both hands on hips were assessed, using the Optojump system (Optojump, Microgate, Bolzano, Italia [33]. The jump height was used for analysis. The 20 m zig-zag test was conducted to measure the participants’ COD performance, using photocell timing gates (Photocells, Brower Timing System, USA) with a protocol described elsewhere [34]. The best time in seconds was used for further analysis. A 30 m linear sprint test was executed using three pairs of photocell timing gates (Photocells, Brower Timing System, UT, USA). Three maximal trials were performed, and the best time was used for analysis. Furthermore, an RSA protocol was conducted using two pairs of photocell timing gates (Photocells, Brower Timing System, UT, USA). The running anaerobic sprint test (RAST) test was conducted. This test consisted of six 35 m linear sprints, interspersed by 10 s of recovery. The best time to complete the test, peak power, and fatigue index measures were used for analysis [35]. The minimum and maximum peak power and the fatigue index were determined using the following equations [36]:

$$\mathrm{Power}=\frac{\mathrm{Weight}\;\times \;{\mathrm{Distance}}^{2}}{{\mathrm{Time}}^{3}}\;\mathrm{and}\;\mathrm{Fatigue}\;\mathrm{Index}=\frac{{\mathrm{Max}}_{\mathrm{Power}}-{\mathrm{Min}}_{\mathrm{Power}}}{\mathrm{Sum}\;\mathrm{of}\;6\;\mathrm{sprints}\;\left(s\right)}.$$

Lastly, the participants completed the YYIR test to measure the VO_2_max. All player had to run 20 m from cone A to cone B and return to cone A (total: 40 m). After every 40 m covered, a 10 s recovery period was ensured. The speed started at 10 km/h, following progressive increases in velocity throughout the test. The YYIR ended when the player achieved total exhaustion or did not reach one of the 20 m cones at the beep timing. The number of completed shuttles and the total distance covered were recorded [37]. Additionally, during the YYIR test, all players used individual Bluetooth HRsensors for heart rate monitor (Polar H10, Polar-Electro, Kempele, Finland, recorded in 5 s intervals) to quantify each athlete’s heart rate maximum (HRmax).

### 2.4. Training Load Monitoring

For measuring the internal load, 10 to 30 min after each training session, all players were asked about how hard the training session was, scored from 1–10, were 1 corresponds to “very light activity” and 10 corresponds to “maximal exertion” [38]. These scores were based on the CR-10 Borg scale [23]. All players were previously familiarized with this daily practice. The collected scores were then multiplied by the total duration in minutes of each training session, to obtain the session RPE [23]. The session RPE for each training session was used as the final outcome for further analysis.

### 2.5. Statistical Analysis

Subjects’ characteristics are presented as means and standard deviations of the variables. For the variables of fitness assessment, a one-way repeated-measure analysis of variance (ANOVA) was performed to clarify the differences among the three assessments. If there was a significant effect, we used the Bonferroni multiple comparison test to determine significant differences among the three conditions for each variable. Eta squared (η^2^) values were used as an indicator of effect size. An η^2^ value of 0.00–0.19 was considered trivial, 0.20–0.49 was small, 0.50–0.79 was moderate, and ≥0.80 was large [39]. The strength of the relationship between the variables of the fitness assessment and accumulated training load was determined using a Pearson product moment linear correlation coefficient (*r*). A paired *t*-test was used to compare the training load between the periods (preseason and midseason). Statistical analyses were conducted using the Statistical Package for the Social Sciences (SPSS version 22.0; Chicago, IL, USA), with a significance level of 0.05.

## 3. Results

The one-way repeated ANOVA revealed no significant differences for any of the variables analyzed at the three moments of fitness assessment (Table 1). As there were no significant changes in the three moments observed, we chose to use the mean as a representative measure of physical status. The *t*-test revealed no differences in the training load between the periods of the season (*t* = 1.216; *p* = 0.235).

**Figure 2:**
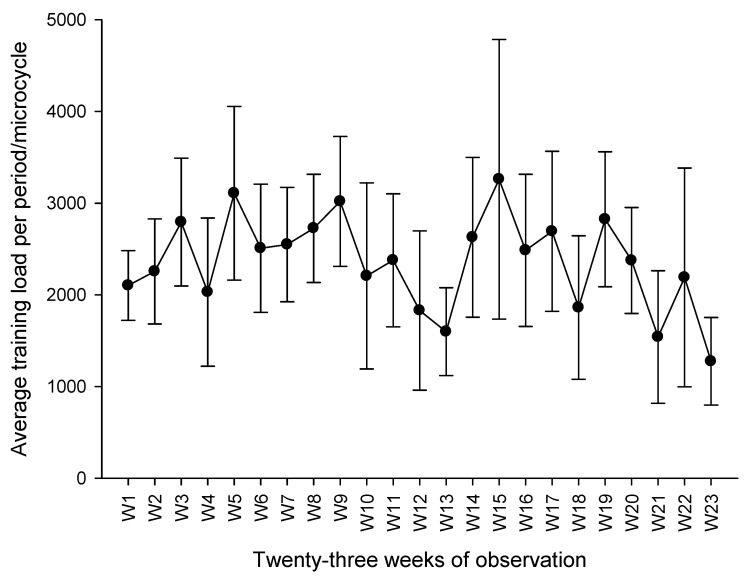
Average training load in the 23 weeks of observation.

The time-course of the training load accumulated in the different microcycles is shown in Figure 2.

Correlations between fitness variables and average training load can be observed in Table 2.

## 4. Discussion

The current study aimed to analyze the variations of fitness status in women soccer players over time (repeated measures) and test the relationships between accumulated training load and fitness variations. To the best of our knowledge, this is the first study to simultaneously analyze variations in fitness status and training load from the beginning of preseason to the end of midseason, in the context of women’s soccer. Concerning the first aim, there were no differences in fitness status during the analyzed period, contrary to our original hypothesis. Furthermore, no significant relationships were observed between the fitness status and the accumulated training load, which is also contrary to our hypothesis.

The literature has shown that athletes generally change their fitness status over the season, although this is not so straightforward. For example, a study showed that players’ aerobic capacity was higher in the midseason than in the pre- and postseason, indicating that the participants tend to reach a peak performance in this variable in the middle of the competitive schedule before it decreases over the subsequent weeks [17]. However, another study revealed that these changes can be very different from season to season [40]. Furthermore, physical performance changes throughout a soccer season can be dependent on the fitness status observed at the beginning of the preseason period [41]. Furthermore, the abovementioned study revealed a lack of positive changes after a preseason period [41].

Similar results were observed regarding VO_2_max, 15 m sprint, and agility tests in another study [10]. On the other hand, [1] found no differences in performance in the countermovement jump with arm-swing and sprint performance over a season, similar to the current results. In addition, previous studies showed that training loads can vary between the different periods of a season [14,15], which suggests that variations in fitness status could be related to the training loads that the players experience.

However, in the current study, there were no significant differences in the training load when comparing the preseason and the midseason, which might justify the absence of differences in players’ fitness status. In fact, there is a need to respect the training principles, such as progressive overload, individualization, and variation for ensuring training adaptations [42]. Indeed, training variation assumes an important role for avoiding a monotonous training cycle and allowing supercompensation to occur [43,44]. For those reasons, the lack of differences in training load during the period observed in the present study may be related to poor management of training loads, as well as poor micro- and mesocycle planning [41]. Therefore, a dose–response relationship between training load variations and fitness status changes in the season could be suggested, although additional studies are required to confirm such an assumption. Specifically, studies in which the training load is consciously manipulated to generate different magnitudes of changes are welcome.

Concerning the associations between physical status and training load, no significant values were reported. The literature suggests that a higher accumulated match time during the season is linked to better speed and CMJ performance, while a higher total exposure time is related to decreased power performances [19]. Therefore, it appears that both training and competitions define soccer players’ physical status. However, another study showed significant negative associations between sRPE and physical fitness changes after a 9 week training period [21]. In fact, corroborating the abovementioned statement regarding the progressive overload training principle need for adaptations to occur, a study conducted on 34 junior male soccer players revealed that elite players with higher perceived training loads throughout a training period presented greater improvements in aerobic performance, when compared with their nonelite counterparts [45]. These facts reinforce the need for a better load management, respecting the training principles and biologic individuality for ensuring positive fitness changes after a training intervention.

At this point, sRPE is postulated as a consistent method of measuring internal training load among sessions for an entire season for youth soccer players [46]. However, sRPE is weakly related to independent high-intensity external load measures [47], indicating that this measure might not capture the complexity of training loads in soccer. Therefore, we suggest that future studies investigate the correlations between fitness status and external training loads in soccer, which could shed light on this topic.

Studies in women’s soccer are still scarce, which justifies one of the strengths of the current research. Moreover, we monitored the players over half a soccer season, which indicates the relevance of the current results to understanding the complexity of the relationship between training loads and fitness status in women’s soccer. However, there is a need for future studies to analyze an entire season, for a greater perception of such relationships.

Nevertheless, caution is required when interpreting the data. First, all athletes in this study belonged to the same team, which reduces the study’s external validity. Additionally, no measures of external training load were taken, which limits the comprehension of the phenomenon. Lastly, we were unable to collect measures of fitness status during the midseason period (just after this stage). For this reason, changes might have occurred that were not captured by our measurements. For those reasons, we recommend future studies to expand the current findings by investigating athletes from a larger sample, collecting external load measures (i.e., high-intensity data), and including more intermediate assessments of fitness status.

## 5. Conclusions

This study revealed no differences in the fitness status of women soccer players during the analyzed season. Moreover, fitness status had no significant relationship with accumulated training load. Further studies should be conducted to identify other possible relationships and eventually determine how specific elements of fitness status are associated with specific efforts exerted during training drills.

## Figures and Tables

**Table 1:** Descriptions, F-statistics, and *p*-values of the fitness variables analyzed at the three moments of fitness assessment.

Variable	M1 (Mean ± SD) M2 (Mean ± SD)	M3 (Mean ± SD)	Mean ± SD		*p*	η2
HRmax (beats/min)	198.2 ± 3.57	201.41 ± 12.80	202.34 ± 8.68	200.65 ± 9.48	0.961	0.03
VO2max mL/(kg·min)	41.21 ± 1.57	43.72 ± 1.57	43.96 ± 1.57	42.96 ± 1.57	0.833	0.07
V10 (m/s)	1.91 ± 0.04	1.84 ± 0.07	1.91 ± 0.15	1.89 ± 0.09	0.564	0.13
V30 (m/s)	4.83 ± 0.09	4.69 ± 0.17	4.81 ± 0.39	4.78 ± 0.20	0.633	0.11
COD20 (s)	5.75 ± 0.09	5.70 ± 0.16	5.83 ± 0.32	5.76 ± 0.20	0.496	0.15
p.max (W)	423.66 ± 50.95	405.12 ± 68.29	403.10 ± 92.00	410.62 ± 64.16	0.365	0.13
p.min (W)	271.43 ± 34.30	266.83 ± 33.77	246.76 ± 50.92	264.87 ± 37.79	0.219	0.13
FI (%)	4.91 ± 1.40	4.03 ± 1.48	5.12 ± 1.81	4.68 ± 1.49	0.768	0.04
SJ (cm)	24.01 ± 2.14	25.03 ± 4.49	25.56 ± 3.68	24.85 ± 3.29	0.684	0.21
CMJ (cm)	25.20 ± 2.43	26.22 ± 4.29	26.29 ±3.39	25.89 ± 3.29	0.179	0.23
YYIR (m)	687.40± 168.93	943.33± 138.33	714.2 ± 163.17	781.67± 210.1	0.095	0.27
Addu (kg)	35.95 ± 7.07	33.51± 7.81	32.69± 6.10	34.03 ± 7.45	0.220	0.15
Abdu (kg)	34.32 ± 5.71	30.92 ± 5.32	32.1 ± 6.16	32.45 ± 6.16	0.561	0.12

M1, M2, and M3: three measurement moments; *p*: *p*-value of F-statistic; η^2^: eta squared values; HRmax: heart rate maximum; VO_2_max: maximum oxygen volume; V10: 10 m sprint; V30: 30 m sprint; COD20: 20 m zig-zag test; p.max: maximum power; p.min; minimum power; FI: fatigue index; SJ: squat jump; CMJ: countermovement jump; YYIR: Yo-Yo intermittent recovery test; Addu: adductors; Abdu: abductors.

**Table 2:** Correlations between mean values fitness and average training load.

Variable	*r*	*p*-Value	*r*	*p*-Value
HRmax	−0.126	0.585	−0.447	0.048
VO_2_max	−0.042	0.850	−0.157	0.486
V10	−0.187	0.417	0.056	0.816
V30	−0.123	0.596	0.023	0.922
COD20	−0.091	0.695	−0.225	0.341
p.max	0.249	0.276	0.058	0.808
p.min	0.351	0.119	0.256	0.276
FI	0.080	0.731	−0.104	0.662
SJ	0.314	0.166	0.330	0.156
CMJ	0.351	0.119	0.441	0.052
YYIR	−0.059	0.811	−0.261	0.295

HRmax: heart rate maximum; VO_2_max: maximum oxygen volume; V10: 10 m sprint; V30: 30 m sprint; COD20: 20 m zig-zag test; p.max: maximum power; p.min; minimum power; FI: fatigue index; SJ: squat jump; CMJ: countermovement jump; YYIR: Yo-Yo intermittent recovery test.
